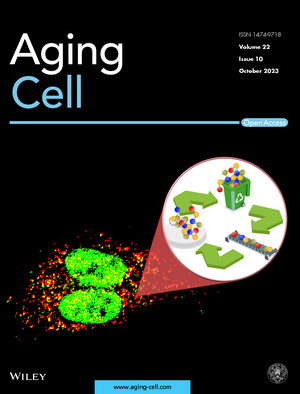# Featured Cover

**DOI:** 10.1111/acel.14016

**Published:** 2023-10-16

**Authors:** Hasan Ishtayeh, Margarita Galves, Tania T. Barnatan, Yevgeny Berdichevsky, Fatima Amer‐Sarsour, Metsada Pasmanik‐Chor, Itzhak Braverman, Sergiu C. Blumen, Avraham Ashkenazi

## Abstract

Cover legend: The cover image is based on the Research Article *Oculopharyngeal muscular dystrophy mutations link the RNA‐binding protein HNRNPQ to autophagosome biogenesis* by Hasan Ishtayeh et al., https://doi.org/10.1111/acel.13949